# A De Novo Loss-of-Function *NCKAP1* Variant in a Boy with Neurodevelopmental Delay and Congenital Heart Defect

**DOI:** 10.3390/children12121680

**Published:** 2025-12-10

**Authors:** Wenying Zhang, Teresa A. Duffy, Cassandra Conrad

**Affiliations:** 1 Department of Pediatrics, University of Cincinnati College of Medicine, Cincinnati, OH 45267, USA; 2 Division of Human Genetics, Cincinnati Children’s Hospital Medical Center, Cincinnati, OH 45229, USA; 3 Division of Developmental and Behavioral Pediatrics, Cincinnati Children’s Hospital Medical Center, Cincinnati, OH 45229, USA

**Keywords:** neurodevelopmental disorders, congenital heart defect, exome sequencing, *NCKAP1*

## Abstract

**Background:** Neurodevelopmental disorders (NDDs), such as autism spectrum disorder (ASD), intellectual disability (ID), and global developmental delay (GDD), frequently have underlying genetic causes. *NCKAP1*, a gene essential for actin cytoskeleton remodeling and neuronal development, has recently gained recognition as a promising candidate gene in NDDs. While not yet linked to a defined Mendelian disorder, damaging *NCKAP1* variants have been identified in individuals with NDDs. *NCKAP1* is also expressed in cardiac tissue, with emerging evidence supporting its potential involvement in cardiac development. Here, we present a case of a patient with neurodevelopmental delay and congenital heart disease (CHD) harboring a novel damaging *NCKAP1* variant. **Methods:** Comprehensive clinical evaluations and trio exome sequencing (proband and parents) were conducted on a patient with complex cardiac and neurodevelopmental phenotypes. **Results:** We identified a de novo heterozygous frameshift variant in *NCKAP1*, NM_205842.3:c.2956_2959del p.(Ser986Hisfs*34), predicted to result in loss of function through nonsense-mediated mRNA decay. The patient’s clinical features included neonatally diagnosed and surgically repaired infradiaphragmatic total anomalous pulmonary venous return (TAPVR), intellectual disability, speech delay, and autistic traits. His NDD phenotypes and variant type align well with previously described *NCKAP1*-associated NDD, while the cardiac anomaly adds evidence to the gene’s expanding phenotypic spectrum. This represents the fourth reported case linking *NCKAP1* variants to CHD and/or neurodevelopmental delay. **Conclusions:** This case strengthens the growing recognition of *NCKAP1* in both neurodevelopment and cardiac formation. It highlights the importance of genetic testing for individuals with overlapping developmental and cardiac conditions. Further research is warranted to elucidate the role of *NCKAP1* in cardiac development and its contribution to CHD.

## 1. Introduction

Neurodevelopmental disorders (NDDs), including autism spectrum disorder (ASD), intellectual disability (ID), and global developmental delay (GDD), as well as neuropsychiatric disorders, affect a significant proportion of children/adolescents and are frequently associated with underlying genetic etiologies [1,2]. Recent advances in next-generation sequencing (NGS) technologies, particularly exome and genome sequencing, have enabled the identification of numerous genes implicated in NDDs, facilitating earlier and more precise diagnoses.

Among these, *NCKAP1* (NCK-associated protein 1) has emerged as a gene of interest due to its critical role in actin cytoskeleton remodeling, a process essential for neuronal migration, dendritic spine formation, and synaptic plasticity [3]. *NCKAP1* encodes a regulatory component of the WAVE2 complex, which mediates actin polymerization in response to RAC1 signaling [4,5,6]. Disruption of this pathway has been linked to impaired neurodevelopment and altered synaptic connectivity [7,8].

Although *NCKAP1* is not currently associated with a defined Mendelian disorder in OMIM (OMIM ID: 604891), several studies have reported deleterious de novo and inherited variants in individuals with ASD, ID, and other neurodevelopmental phenotypes [9,10]. Notably, most reported cases involve truncating or frameshift variants predicted to result in loss-of-function through nonsense-mediated mRNA decay. However, the full phenotypic spectrum of *NCKAP1*-related disorders is still being delineated. In addition to its expression in the central nervous system, *NCKAP1* is also expressed in cardiac tissue [7], shown in the GTEx Portal (https://gtexportal.org/home/gene/NCKAP1 (accessed on 4 December 2025)), raising the possibility of a broader role beyond neurodevelopment. However, the evidence for cardiac role is limited to animal studies and three reported cases [9,11,12,13,14,15].

Here, we report a patient with a de novo heterozygous loss-of-function variant in *NCKAP1* who presented with both neurodevelopmental delay and a complex congenital heart disease (CHD), specifically, infradiaphragmatic total anomalous pulmonary venous return (TAPVR). This case adds to the limited but growing evidence suggesting a potential role for *NCKAP1* in cardiac development and underscores the value of comprehensive genetic evaluation in patients with co-occurring NDDs and CHD.

## 2. Case Description

The patient was born at 39 weeks’ gestation, weighing 7 pounds 2 ounces, and was the third child (G3P3). Shortly after birth, he experienced persistent respiratory distress. Echocardiography revealed infradiaphragmatic TAPVR. All four pulmonary veins were draining to confluence. There was a large descending vertical vein with a mean gradient of 14 mmHg as it entered a hepatic vein. There was a moderately sized secundum atrial septal defect, shunting all right to left with a mean gradient across atrial septum of 1.29 mmHg. The right ventricle was severely dilated with normal systolic function. The left ventricle systolic function was mildly diminished.

At seven days of age, he underwent surgical repair with direct anastomosis and atrial septal defect patch closure. An echocardiogram following repair demonstrated the pulmonary venous confluence draining unobstructed to the left atrium. There was a mean 1–2 mmHg gradient at the left atrium confluence.

His postoperative course was without complication, and he began regular follow-up with pediatric cardiology specialists. A renal ultrasound at one year of age showed normal kidneys and bladder.

At 4 years old, the patient was referred to the Division of Developmental and Behavioral Pediatrics (DDBP) as part of a multidisciplinary neurodevelopmental cardiology clinic for evaluation of developmental delay and possible autism spectrum disorder. Clinical history and examination revealed delays across multiple domains, including expressive and receptive language, social communication, gross and fine motor skills, pre-academic abilities, and daily living skills, consistent with global developmental delay (Table 1). While the patient demonstrated some joint attention, gesturing, and eye contact, these were less consistent than expected for his age. Additionally, he exhibited repetitive behaviors, difficulty with transitions, and sensory difficulties warranting further evaluation for possible autism spectrum disorder.

Physical examination noted microcephaly (head circumference = 47.5 cm, 1st percentile) and unique facial features, including protruding ears with cupped earlobes, wide-set narrow eyes, and small teeth. Echocardiography demonstrated unobstructed pulmonary venous return to the left atrium with a mean gradient of 1 mmHg, mild aortic root dilation, and normal size and systolic function of both ventricles. A comprehensive psychological evaluation, including formal cognitive, adaptive, and socio-emotional assessment along with specific evaluation for autism spectrum disorder was completed. As measured by the Wechsler Preschool and Primary Scale of Intelligence, Fourth Edition (WPPSI-IV), the patient’s full scale intelligence quotient (FSIQ) fell in the Extremely Low range (FSIQ = 69, 2nd percentile). His adaptive skills, as measured on the Vineland-3, fell in the low range with a composite score of 60 (<1st percentile). Behavioral ratings completed by parents, including the Child Behavioral Checklist, indicated concerns regarding emotional reactivity, withdrawn/depressed behavior, attention problems, and aggressive behaviors. The patient’s performance on an autism specific measure, the Autism Diagnostic Observation Schedule, second edition (ADOS-2), module 1, was varied. He exhibited some symptoms suggestive of possible autism including occasional echoing of speech, unusual sensory interest, and some repetitive behavior. However, he also presented with numerous positive social communication skills including directed and functional vocalizations, directed facial expressions, imitation, shared enjoyment, social referencing, initiation of joint attention, response to joint attention, engagement in interactive play, and emerging functional/creative play skills. The patient’s scored performance fell within an ADOS-2 classification of non-spectrum, which was consistent with the examiner’s clinical impression.

Given the patient’s social strengths, an autism diagnosis was deferred with ongoing monitoring recommended. Subsequently, the patient began physical, occupational, and speech therapies at age 4. Audiological evaluation confirmed normal hearing and ruled out hearing loss as a contributing factor to his speech or developmental delays. The patient continued regular developmental monitoring through DDBP and ongoing cardiology follow-up. At 7 years old, he remained asymptomatic from a cardiac standpoint, with cardiology visits spaced to every 18 months due to stable cardiac status. On echocardiogram at age 7, the pulmonary venous confluence drained unobstructed to the left atrium with a mean gradient of 1.4 mmHg. Left and right ventricles were normal in size with normal systolic function.

At a DDBP visit at age 7, by clinical history and examination he had made interval progress in verbal communication. He predominately communicated verbally in phrases with mostly clear articulation. He followed 1–2 step instructions. He attended school in a special education classroom and received supports through an Individual Education Plan. He was behind grade level academically. He was able to count to 5, trace his name, identify letters, shapes, and colors. He benefitted from support with activities of daily living such as getting dressed. He ambulated independently and used utensils to feed himself. He had a strong social interest and enjoys interactions with adults and older peers, but had some difficulty with interactions with same-aged peers. Results from a school-based psychological evaluation indicated ongoing significant delays in both cognitive and adaptive functioning. On the Woodcock-Johnson Tests of Cognitive Abilities, the patient obtained a Cognitive Composite score of 57, which falls in the extremely low range (below the 1st percentile). Adaptive functioning, as measured by the Adaptive Behavior Assessment System–Third Edition (ABAS-3), revealed composite scores of 70 from the teacher (2nd percentile) and 60 from the parent (<1st percentile), with parent ratings suggesting more pronounced difficulties across home and community settings. Taken together, these findings demonstrated pervasive challenges in cognitive and adaptive domains, consistent with a diagnosis of mild intellectual disability (Table 1).

## 3. Genetic Findings

Genetic testing was arranged at 5 years old and included Fragile X syndrome, SNP microarray, and trio Exome sequencing (proband and both parents). Both Fragile X and microarray yielded negative results.

**Figure 1:**
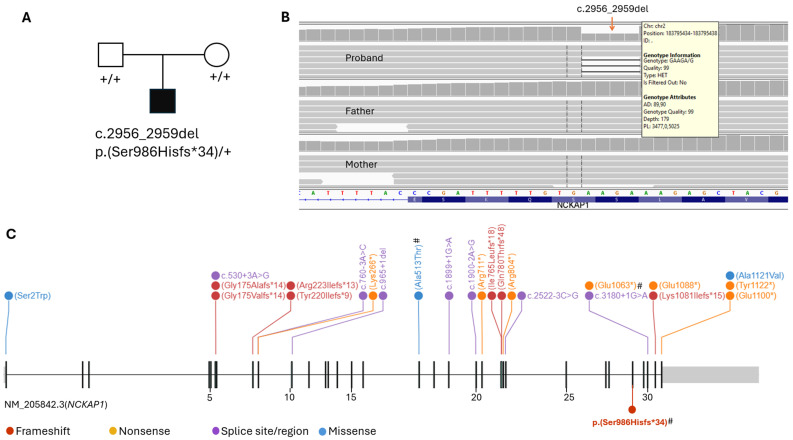
(**A**). Pedigree showing the de novo heterozygous *NCKAP1* variant identified in the proband. (**B**). NGS showing the heterozygous 4 bp deletion in *NCKAP1* in the proband with a variant allele frequency of 0.5, absent in both parents. (**C**). Schematic diagram of *NCKAP1* (NM_205842.3) showing previously reported likely or potentially disruptive single nucleotide variants and small indels associated with NDD and/or CHD (above the gene), and the 4 bp deletion identified in the proband (below, highlighted in bold). Exon numbers are indicated. # denotes variants detected in individuals in whom a cardiac phenotype was reported. (**C**) was generated using ProteinPaint [16] and subsequently modified for clarity.

Trio Exome sequencing identified a heterozygous frameshift variant in *NCKAP1* (NM_205842.3), c.2956_2959del p.(Ser986Hisfs*34), in the proband. This 4-base-pair deletion is in exon 28 of 32 and is predicted to cause a frameshift starting at amino acid position Ser986, leading to a premature translation termination at 34 codons downstream. This variant is expected to result in either a truncated protein or loss of protein due to nonsense-mediated mRNA decay. Neither parent carries the variant, suggesting it is most likely de novo (Figure 1A,B). However, the possibility of gonadal mosaicism in one parent cannot be entirely excluded. The results were confirmed by Sanger sequencing (Supplementary Figure S1).

This variant has not been previously reported in ClinVar or published literature and it is absent from population databases such as Genome Aggregation Database (gnomAD). *NCKAP1* is highly constrained and intolerant for loss-of-function variants with a probability of Loss-of-function Intolerance (pLI) of 1 (cutoff > 0.9) and Loss-of-function Observed/Expected Upper Fraction (LOEUF) of 0.07 (90% confidence interval: 0.04–0.14; cutoff < 0.35) in gnomAD. Additionally, no disease phenotype is currently listed for *NCKAP1* in OMIM. Nevertheless, deleterious variants in *NCKAP1* have been identified in individuals with NDDs. Both de novo or inherited disruptive variants in this gene have been reported in families with a dominant inherited pattern [9,10]. Furthermore, truncating variants downstream of this identified variant have been reported as pathogenic or likely pathogenic in ClinVar, including p.(Asn1080Lysfs*54) and p.(Glu1094*) (ClinVar ID: 834055 and 3064095, respectively). This variant, along with previously reported likely or possible disruptive substitutions or small indel variants, is illustrated in the schematic diagram of *NCKAP1* (Figure 1C).

The proband’s phenotype, including intellectual disability, speech delay, global developmental delay, repetitive behaviors, and CHD, is consistent with the phenotypic spectrum associated with pathogenic *NCKAP1* variants [9]. While CHD is not a commonly reported feature of *NCKAP1*-related disorders, it has been described as early as 2013 in one individual with left ventricular outflow tract obstruction (LVOTO), who carried a de novo nonsense variant (p.(Glu1063*), reported as p.(Glu1057*) in Zaidi et al., 2013 [15]). In addition, Guo et al. (2020) reported two individuals with co-occurring heart defects and NDDs, further supporting a potential [9] (Supplementary Table S1).

Based on the available information, this variant identified in our patient is classified as likely pathogenic (PVS1-strong, PS2-supporting, PM2-supporting, with one strong, and two supporting criteria based on the updated ACMG criteria from ClinGen Sequence Variant Interpretation Working group (https://clinicalgenome.org/working-groups/sequence-variant-interpretation/ (accessed on 03/11/2025)) [17]. To date, this represents the fourth reported case of a likely disruptive *NCKAP1* variant in a patient presenting with congenital heart defect and/or neurodevelopmental delay.

Given the de novo status of the variant and the uncertain cardiac penetrance, the recurrence risk for future pregnancies is expected to be low (<1%) but not zero, due to the possibility of parental gonadal mosaicism. The family was advised to continue developmental surveillance, therapies, and educational supports in accordance with guidelines from the American Heart Association (AHA), the American Academy of Pediatrics (AAP) and the Cardiac Neurodevelopmental Outcome Collaborative (CNOC) for children with cardiogenetic conditions [18,19,20]. To promote transparency and contribute to shared clinical genetic knowledge, we will deposit this variant in ClinVar.

## 4. Discussion

NCKAP1 is one of the regulatory proteins of the Wiskott-Aldrich syndrome protein-family verprolin-homologous protein-2 (WAVE2) complex. As a core component of the WAVE2 complex, alongside WAVE2 (encoded by *WASF2*), BRK1, CYFIP1, and ABI1, NCKAP1 mediates signaling from small GTPase to promote actin polymerization [4,5]. Specifically, it is regulated by RAC1 to facilitate branched actin synthesis, essential for proper cytoskeleton organization [6]. WAVE2 complex proteins are expressed abundantly and mediate various cellular events in the brain [5], such as neurogenesis and neuronal migration, neurite and axonal outgrowth, synapse formation and plasticity, oligodendrocyte morphogenesis and axonal myelination, and microglia migration and phagocytosis. Although the role of the WAVE2 proteins in cardiogenesis is limitedly understood, cardiac anomalies have been observed in animal models: mice with WAVE2 gene knockout and zebrafish with CRISPR-mediated *wasf2, brk1*, and *nckap1* knockdown exhibited structural heart defects [11,12,13,14].

NCKAP1 is particularly crucial in the developing nervous system, where it contributes to neuronal migration, dendritic spine formation, and synaptic plasticity-processes integral to cognitive development and neural connectivity [3]. Experimental knockdown of *NCKAP1* using antisense oligonucleotides has been shown to induce apoptosis in neuronal cells [7] and its disruption impairs neuronal migration [8].

Three likely disruptive de novo variants in *NCKAP1*, p.(Glu1088*), p.(Gly175Valfs*14), and c.530+3A>G, have been identified repetitively in three patients with ASD (patient ID: 12764, 14030, and 14484) across multiple studies from 2012 to 2019 [21,22,23,24,25]. However, detailed clinical data for these cases were not provided. An additional de novo splice site variant, c.3180+1G>A, was reported in 2016 in another autism study but with no details provided as well [25].

In a large multigenerational family, Anazi et al. (2017) identified a heterozygous truncating variant, p.(Glu1100*), in *NCKAP1* segregating with autosomal dominant, non-syndromic mild ID [26]. The proband presented with tip-toe gait, ID, speech and language delay, attention deficit hyperactivity disorder (ADHD), hypertelorism, macrocephaly, and tall stature. The variant was inherited from her affected father and detected in all seven affected family members tested across three generations.

Through an international collaboration, Guo et al. (2020) described 21 affected individuals with detailed clinical information from 20 unrelated families harboring predicted deleterious variants in *NCKAP1* [9]–13 de novo, 5 inherited, and 2 of unknown inheritance. Most variants were protein-truncating and predicted to undergo nonsense-mediated mRNA decay, though one case involved a 240 kb deletion and another a chromosomal inversion with a breakpoint occurred in the first intron of *NCKAP1*. The predominant phenotype across this cohort was neurodevelopmental, including autistic features (73%), speech-language problems (74%), motor delay (73%), and ID or learning disabilities (63%). Additional commonly reported features included repetitive behaviors (69%), aggressive behavior (57%), ADHD (54%), sleep disturbance (60%), seizure (41%), tall stature (38%), overweight (25%), and gastrointestinal disturbances (25%).

Importantly, Guo et al. (2020) also documented cardiac abnormalities in two unrelated families, one with bicuspid aortic valve (BAV) harboring a de novo gross chromosomal inversion interrupting *NCKAP1*, and another with dilated cardiomyopathy (DCM) and left ventricular systolic dysfunction and harboring a de novo missense variant, p.(Ala513Thr) [9]. In 2013, a de novo nonsense variant, p.(Glu1063*), was reported in a patient with LVOTO [15]. Including the current case, who had infradiaphragmatic TAPVR, there are now four reported cases of de novo likely deleterious *NCKAP1* variants associated with CHD and/or NDD (Supplementary Table S1).

Although the number of reported cases with CHD is small and these four cases represent three different biological mechanisms: outflow tract patterning (the BAV and LVOTO cases), pulmonary venous incorporation (our case), cardiomyocyte structural integrity (the DCM case), both clinical observations and animal model data suggest a possible role for *NCKAP1* in cardiac development. The evidence is still preliminary, and the apparent penetrance is low [9], but the findings are promising. Additional studies are needed to strengthen the evidence and clarify the gene’s potential contribution to CHD.

Congenital heart defect is the most common and clinically devastating birth defect and a leading cause of infant mortality. Advances in surgical interventions have significantly improved survival, with >90% of children with CHD survive to adulthood [27]. Nonetheless, children with CHD are at increased risk of developmental delay, ID, or ASD [19,20]. In recognition of this, the AHA, AAP and CNOC recommend regular neurodevelopmental evaluations, as the child ages, to identify and address developmental, learning, and behavioral concerns [18,19,20].

The growing accessibility of NGS has greatly enhanced our understanding of the genetic contributions to both NDDs and CHD. This case further underscores the importance of integrating genetic evaluation—including testing—into the routine medical assessment of children with NDD and/or CHD.

## 5. Conclusions

We report a patient with a heterozygous de novo loss-of-function frameshift variant in *NCKAP1*, NM_205842.3:c.2956_2959del p.(Ser986Hisfs*34), who presented with CHD (infradiaphragmatic TAPVR) and developmental delay. This case represents the fourth report of a de novo *NCKAP1* likely pathogenic variant in a patient with CHD and/or NDD. These findings contribute to the emerging evidence linking *NCKAP1* loss-of-function variants to neurodevelopmental and, to a lesser extent, cardiac phenotypes. Given the common co-occurrence of NDDs and CHD, our report highlights the value of comprehensive genetic evaluation as part of routine care. Early molecular diagnosis may facilitate personalized care strategies and support better developmental outcomes for affected children.

## Figures and Tables

**Table 1:** Developmental, Cardiac, and Neuropsychological Trajectory Across Ages 4 and 7 Years Old.

Age	Motor Skills	Language/Communication	Activities of Daily Living (ADLs)	Echocardiography	Cognitive & Adaptive Testing
**4 years**	Delays in gross and fine motor skills; required physical and occupational therapy	Delays in expressive and receptive language; inconsistent joint attention, gesturing, and eye contact; occasional echolalia	Delays in daily living skills; required support with dressing, feeding, and pre-academic abilities	Unobstructed pulmonary venous return to the left atrium with a mean gradient of 1 mmHg, and normal size and systolic function of both ventricles.	**WPPSI-IV:** FSIQ = 69 (Extremely Low, 2nd percentile). **Vineland-3:** Adaptive Composite = 60 (<1st percentile).
**7 years**	Ambulates independently; uses utensils; improved fine motor skills (tracing name, identifying shapes/letters)	Communicates verbally in phrases with mostly clear articulation; follows 1–2 step instructions; strong social interest but difficulty with peers	Able to dress with support; feeds self; behind grade level academically but demonstrates progress in counting, tracing, and recognition tasks	Unobstructed pulmonary venous return to the left atrium with a mean gradient of 1.4 mmHg, and normal size and systolic function of both ventricles	**Woodcock-Johnson:** Cognitive Composite = 57 (Extremely Low, <1st percentile). **ABAS-3:** Teacher Composite = 70 (2nd percentile); Parent Composite = 60 (<1st percentile).

ABAS-3: the Adaptive Behavior Assessment System, Third Edition; Vineland-3: the Vineland Adaptive Behavior Scales, Third Edition; Woodcock-Johnson: the Woodcock-Johnson Tests of Cognitive Abilities; WPPSI-IV: the Wechsler Preschool and Primary Scale of Intelligence, Fourth Edition.

## Data Availability

The original contributions presented in the study are included in the article, further inquiries can be directed at the corresponding author.

## Supplementary Materials

The following supporting information can be downloaded at https://www.mdpi.com/article/10.3390/children12121680/s1, Figure S1: Chromatogram showing the heterozygous 4 bp deletion in *NCKAP1* in the proband, absent in both parents, confirmed by reverse Sanger sequencing reads; Table S1: The *NCKAP1* variants and cardiac findings in patients and animal models.
