# Supplementary material for: A De Novo Loss-of-Function NCKAP1 Variant in a Boy with Neurodevelopmental Delay and Congenital Heart Defect
Source: Children (Basel). 2025 Dec 10;12(12):1680. doi: 10.3390/children12121680 (PMC12731551; doi:10.3390/children12121680)
Supplement: Supplementary file 1 [file children-12-01680-s001.zip › children-3999357-supplementary.pdf]

Supplementary Table S1. The *NCKAP1* variants and cardiac findings in patients and animal models.

		This study	Zaidi et al., 2013 and Edwards et al., 2020	Guo et al., 2020	Guo et al., 2020	Rakeman et al., 2006		Edwards et al., 2020
	features	This family	Patient (1-01360)	Patient (19)	Patient (17)	mice-*Nap1*^{khlo}	mice-*Nap1*^{GT}	zebrafish model
variant	DNA change	c.2956_2959del	c.3187G>T	c.1537G>A	inv(2)(2pter->p23.1(30,118,062)::2q32.1(183,031,933)->2p23.1 (30,118,062)::2q32.1(183,031,934)->2qter)	c.50T>C	a gene-trap insertion in *Nap1* intron 24, which encodes a truncated protein fused to β-geo.	CRISPR-mediated knockdown of *nckap1*
	protein change	p.(Ser986Hisfs*34)	p.(Glu1063*)	p.(Ala513Thr)	p.(?)	p.(Leu17Pro)	p.(?)	p.(?)
	variant type	frameshift	nonsense	missense	gross chromosomal inversion interrupting *NCKAP1* (one breakpoint in first intron)	loss of function (strong or null)	loss of function (weak)	loss of function
	inheritance	de novo	de novo	de novo	de novo	-	-	-
	gnomAD.v4	absent	absent	0.00006% (1 heterozygote)	absent	absent	-	-
	ClinVar	absent	absent	absent	absent	absent	-	-
demographic	age	newborn	NA	2 years old	9 years old	-	-	-
	sex	male	NA	female	male	-	-	-
phenotypes	cardiac phenotype	TAPVR	LVOTO (bicommissural (bicuspid, bileaflet, bifolicate) aortic valve |coarctation |left aortic arch)	DCM, left ventricular systolic dysfunction	BAV	fail to form a single heart tube (cardia bifida)	fail to form a single heart tube (cardia bifida)	reversed heart looping observed in 16/93 F0 zebrafish (19%)
	brain phenotype or MRI	NA	NA	normal	Small bilateral pretemporal arachnoid cysts of doubtful clinical significance. Otherwise normal MRI of the brain without contrast.	fail to close the neutral tube	fail to close the neutral tube	not observed
	Motor delay	yes, in both gross and fine motor skills	NA	yes	yes	-	-	-
	Regression of developmental milestones	NA	NA	Some gross motor regression around 2 1/2 years old (was able to use stairs	no	-	-	-

				and then started falling down stairs)				
	Intellectual disability	mild	NA	NA	yes	-	-	-
	speech-language problems	yes	NA	previously in ST but test out	yes	-	-	-
	behavior issues	repetitive behavior, sensory difficulties, difficulty with transition, social communication issue; concerns for emotional reactivity, withdrawn/depressed behavior, attention problems, and aggressive behaviors	NA	NA	easily angered, tantrums; aggressive behavior; anxiety (with sleep)	-	-	-
	autism	autistic trait (occasional echoing of speech, unusual sensory interest, and some repetitive behavior) but also presented with numerous positive social communication skills.	NA	no formal evaluation	no (In 2017, he was evaluated with ADOS2, module 3. He did not meet criteria for autism spectrum disorder and was noted to have a number of social-communicative strengths, including use of gestures, eye contact, and ability to engage in back and forth conversation.)	-	-	-
	microcephaly	<1st percentile	NA	NA	no	-	-	-
	facial deformity	protruding ears with cupped earlobes, wide-set narrow eyes, and small teeth	NA	NA	short forehead, low hairline, bushy eyebrows, slightly downturned palpebral fissures, thick lashes, broad nose	-	-	-
	Other symptoms	NA	NA	poor weight gain; low tone; fusion of T8 through T10 with a mild focal lordosis, thoracolumbar scoliosis, history of torticollis	1 lipoma, 1 café au lait macule, pityriasis alba; history of headache	-	-	-

ADOS2: the Autism Diagnostic Observation Schedule, Second Edition; BAV: bicuspid aortic valve; DCM: dilated cardiomyopathy; LVOTO: left ventricular outflow tract obstruction; NA: not available; ST: speech therapy; TAPVR: total anomalous pulmonary venous return

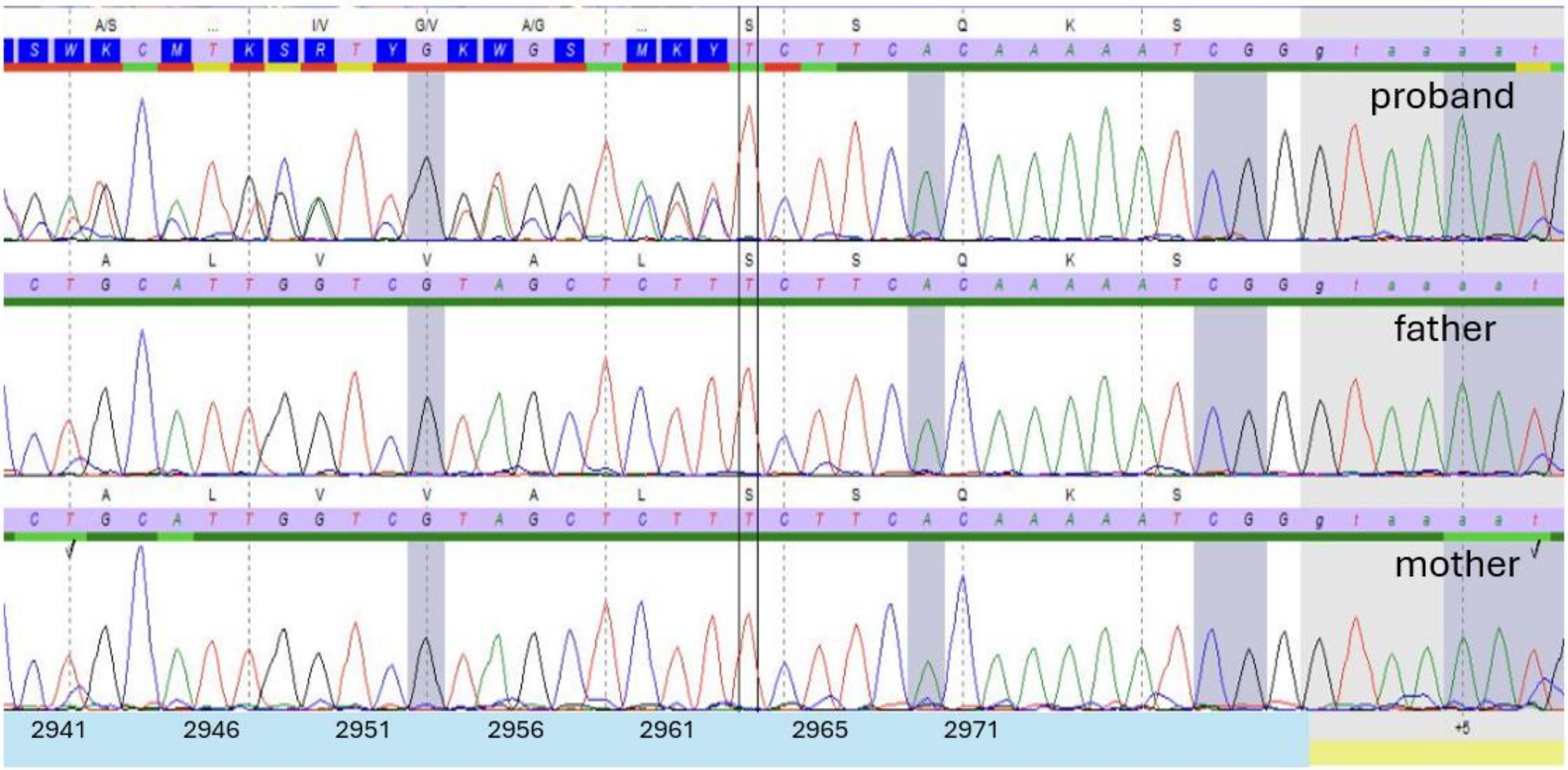

NCKAP1 E28

Supplementary Figure S1. Chromatogram showing the heterozygous 4-bp deletion in *NCKAP1* in the proband, absent in both parents, confirmed by reverse Sanger sequencing reads.
